# Establishing the Resilient Under-resourced Regions Addressing Life-threatening risk in VETeranS dataset (RURAL-VETS): building insights and critical data access for rural-residing Veterans

**DOI:** 10.3389/fpubh.2026.1912223

**Published:** 2026-09-09

**Authors:** Ryan Holliday, John R. Blosnich, Emily R. Edwards, Matthew J. Igo, Claire A. Hoffmire, Christina R. LaValle, Lindsey L. Monteith, Lisa A. Brenner

**Affiliations:** 1 VA Pacific Islands Health Care System, Honolulu, HI, United States; 2 VA Eastern Colorado Health Care System, Aurora, CO, United States; 3 University of Colorado Anschutz Medical Campus, Aurora, CO, United States; 4 Suzanne Dworak-Peck School of Social Work, University of Southern California, Los Angeles, CA, United States; 5 Center for Healthcare Evaluation, Research, and Promotion, VA Pittsburgh Healthcare System, Pittsburgh, PA, United States; 6 VA VISN 2 MIRECC, James J. Peters VA Medical Center, Bronx, NY, United States; 7 Icahn School of Medicine at Mount Sinai, New York, NY, United States

**Keywords:** data integration, rural, suicide prevention, Veteran, Veterans Affairs (U.S.)

## 1 Introduction

Despite being an area of focus for prevention, elevated risk for suicide among rural-residing^1^ Veterans has persisted for nearly two decades (1). In the most recent Department of Veterans Affairs (VA) Suicide Prevention Report, the 2023 suicide rate among rural-residing Veterans was 45.1 per 100,000, exceeding that of 38.9 per 100,000 among Veterans residing in more urban areas (2). To address this health disparity, the VA has implemented patient and community-based initiatives focused on preventing rural Veteran suicide, such as the expansion of telehealth services (3) and *Together with Veterans*, a community-based suicide prevention program for rural-residing Veterans (4). Some of these prevention efforts have focused on increasing access to evidence-based, potentially life-saving interventions [e.g., Cognitive Behavioral Therapy for Suicide Prevention (CBT-SP); (5)] and/or prevention strategies [e.g., lethal means safety (LMS); (6)]. Nonetheless, rural mental health, including suicide prevention, remains severely under-researched (7). Additional work remains necessary to comprehensively understand the intrapersonal, interpersonal, and community-based risk and resilience factors unique to rural-residing Veterans.

Indeed, the experiences of rural-residing Veterans are often unique, attesting to their need for specialized consideration. For example, in comparison to urban- or suburban-residing individuals, those in rural regions have decreased access to specialty healthcare to treat complex, chronic health [e.g., cardiovascular disease; traumatic brain injury (TBI)-related sequelae] and psychiatric conditions [e.g., depression, posttraumatic stress disorder; (8, 9)], social resources and services [e.g., transitional housing, food pantries; (10)], and interpersonal and community-based supports (e.g., further distances to friends and family; long drives to values-based volunteer work). Individuals in rural regions also have greater access to more lethal means of suicide, such as firearms (11). These potential vulnerabilities—individually and in combination (12)—may explain why suicide mortality is higher among rural-residing Veterans (2), despite having a similar prevalence of suicidal ideation and non-fatal suicide attempts as Veterans residing in urban areas (13). However, the question of “which Veterans?” are at greatest risk, in addition to “when” this risk is greatest and therefore necessitating increased care, continues to vex community members, Veterans, family members, providers, researchers, and policy makers.

## 2 Discussion

**Figure 1:**
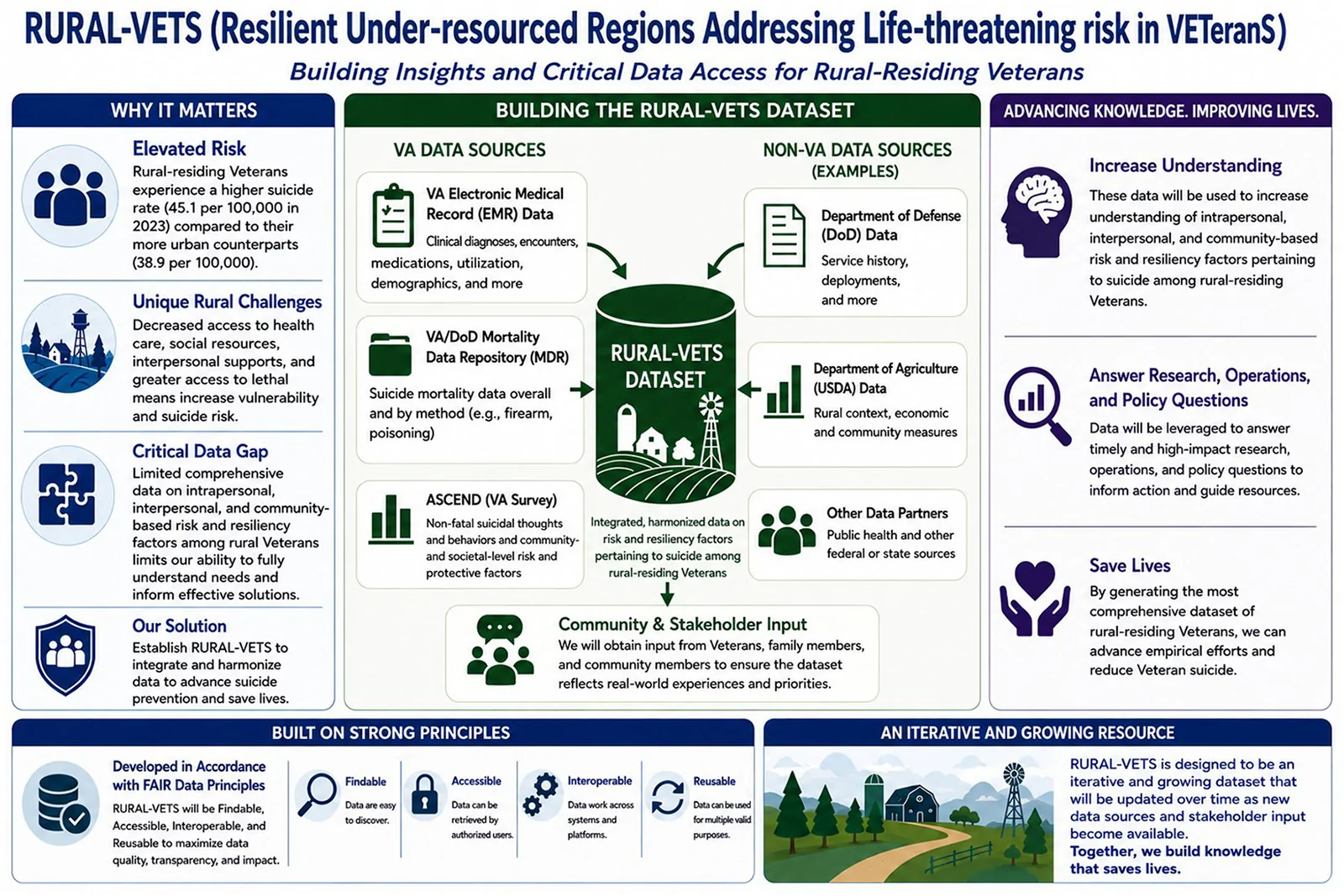
Graphical depiction of the proposed RURAL-VETS dataset.

It is the opinion of the authors that a lack of a cultivated, comprehensive dataset focused on intrapersonal, interpersonal, and community-based risk and resilience factors among rural-residing Veterans poses a barrier to increasing understanding of potential treatment/intervention targets. This is especially important as rural regions are notably heterogeneous which can impact large-scale evaluations (14). More specifically, rural regions can vary based on social determinants, including access to health and social services, as well as contextual factors (e.g., geographic barriers to transportation). These factors can further extend to Veterans, including heterogeneity based on demographic (e.g., age, era served) and access factors. Given this, harmonizing and curating data that spans myriad factors is an important next step to ensure the VA can work with rural community members to decrease risk for suicide and other associated and preventable causes of death [e.g., overdose; (15)]. To address current knowledge gaps, our team is constructing the RURAL-VETS (*Resilient Under-resourced Regions Addressing Life-threatening risk in VETeranS*) dataset (see Figure 1).

### 2.1 Data sources and integration

Initially, members of the RURAL-VETS team will synthesize VA electronic medical record data with information from the VA/DoD Mortality Data Repository (MDR), which aligns with ongoing VA efforts to track suicide prevalence and risk among rural-residing Veterans (2). However, we will expand upon these efforts by harmonizing the RURAL-VETS dataset with information regarding Veterans *not* using VA healthcare. Combining MDR data with survey data from the VA's non-fatal suicidal self-directed violence (NF-SSDV) surveillance system [i.e., Assessing Social and Community Environments with National Data (ASCEND) for Veteran Suicide Prevention; (16)] would be valuable, given this nationally representative dataset measures self-reported suicidal ideation and NF-SSDV, as well as community- and societal-level risk and protective factors. Data from additional non-VA data sources (e.g., Departments of Defense or Agriculture, Harvard University's Social Capital Atlas, National Neighborhood Data Archive, Association of Religious Data Archives) will also be considered, which has the ability to augment efforts to characterize not only community-based risk factors (e.g., food insecurity rates or recent natural disasters in a specific community), but also resilience factors that VA can leverage for prevention efforts.

Synthesizing these diverse datasets can be complex, especially in instances in which data cannot be linked based on a highly specific individual-level identifier (e.g., social-security number). To address these barriers, our team has developed linkage processes that identify shared variables across datasets [e.g., day of month of birth, sex, first initial of last name; (17)]. Even with these strategies, however, we anticipate that some datasets may not be combinable. Such instances will be used to inform VA documentation policies (e.g., adding identifying elements to synthesize non-VA and VA data) to facilitate long-term data integration efforts. Nonetheless, in beginning to combine these datasets, RURAL-VETS has the potential to include identifying social or community resources available to high-risk rural-residing Veterans (e.g., community centers, food pantries, spiritual institutions) that could partner with VA to identify and engage individuals in suicide prevention.

### 2.2 Analytic aims

We believe the RURAL-VETS dataset has the potential to guide understanding of the complex interplay of intrapersonal, interpersonal, and community-based risk and resilience factors driving, or preventing, suicide. Initial analyses will focus on comparing these factors across rural Veteran groups to characterize specific suicide risk phenotypes based on social determinants of health, health care use, and diagnostic factors (e.g., TBI, psychiatric diagnosis) in the context of individual-level protective (e.g., community cohesion) and risk (e.g., access to firearms) factors. Identification of phenotypes of those at greatest risk for suicide (e.g., rural-residing Veterans experiencing concurrent homelessness and TBI-related sequelae) can be used to inform how to optimize development and/or implementation of life-saving treatment approaches (e.g., concurrent telehealth brain-health-related care and case management). Several analytic approaches can be leveraged to generate phenotypes of rural-residing Veterans. For example, our team has previously utilized latent class analysis and machine learning techniques to translate complex, large data systems into practical clinical tools [see (18, 19)]. Concomitantly, once generated, phenotyping data can inform understanding of which strategies are beneficial in mitigating suicide risk. For example, data can assist in identifying VA services (e.g., transitional housing) or interventions (e.g., CBT-SP, LMS) that may reduce long-term suicide risk. These findings, in turn, can inform which specific services or interventions might warrant additional dissemination and engagement efforts for Veterans in rural regions. Despite this, it is important to note that these assertions are aspirational in nature, and given the complex nature of providing care for rural-residing Veterans, additional research that is complimentary to the RURAL-VETS project is likely needed.

### 2.3 Future of RURAL-VETS and patient-centered engagement

Long-term, data could potentially be leveraged to answer VA research, operational, and policy-related questions with timeliness, efficiency, and validity. In addition, input from Veterans, family members, and community members will be considered (e.g., Veteran engagement boards) throughout the life of the project to ensure integration of pressing questions and concerns of those with lived experience. Additionally, RURAL-VETS is being developed in accordance with FAIR (Findability, Accessibility, Interoperability, and Reusability) data principles (20). While RURAL-VETS is intended to help guide VA operations and research, it will also be designed to further the field of rural health research as a whole. Our team will strive to generate synergy between researchers, both within and outside VA, to ensure that findings are not siloed and that actionable findings with clear clinical implications are widely disseminated. Toward that end, ultimately, we envision RURAL-VETS as a living data repository that will be updated over time as new data become available, to drive research aimed at saving and improving lives of rural-residing Veterans.

## Footnotes

1 Within the VA, “rural” residence is classified based on the Rural-Urban Commuting Area (RUCA) codes system. The RUCA code system was developed by the Department of Agriculture and the Department of Health and Human Services and takes into account population density, as well as how closely a community is linked socio-economically to larger urban centers. RUCA is also used by other federal organizations; for example, the United States Census Bureau, to count rural-residing citizens [see Department of Veterans Affairs (21) for additional information].
